# Phytochemical Screening of Ultrasonic Extracts of *Salix* Species and Molecular Docking Study of *Salix*-Derived Bioactive Compounds Targeting Pro-Inflammatory Cytokines

**DOI:** 10.3390/ijms241411848

**Published:** 2023-07-24

**Authors:** Emilia Gligorić, Ružica Igić, Branislava Teofilović, Nevena Grujić-Letić

**Affiliations:** 1 Department of Pharmacy, Faculty of Medicine, University of Novi Sad, Hajduk Veljkova 3, 21000 Novi Sad, Serbia; 2 Department of Biology and Ecology, Faculty of Sciences, University of Novi Sad, Trg Dositeja Obradovića 3, 21000 Novi Sad, Serbia

**Keywords:** *Salix*, ultrasound-assisted extraction, antioxidant activity, molecular docking, TNF-α, IL-6

## Abstract

Willow bark (*Salix* spp., Salicaceae) is a traditional analgesic and antirheumatic herbal medicine. The aim of this study was to evaluate and compare the phytochemical and antioxidant profiles of leaf and bark extracts of six species of the genus *Salix* obtained by ultrasound-assisted extraction (UAE) and to examine the inhibitory potential of target bioactive compounds against two inflammatory mediators, tumor necrosis factor alpha (TNF-α) and interleukin 6 (IL-6), through in silico molecular docking. The total phenolic and flavonoid content of the extracts was estimated using spectrophotometric methods and the antioxidant activity using 2,2-diphenyl-1-picrylhydrazyl (DPPH^•^) and hydroxyl radical (^•^OH) scavenging assays. Chemical profiling of extracts was carried out using high-performance liquid chromatography coupled with a diode array detector (HPLC-DAD). Principal component analysis (PCA) was performed to differentiate the sample extracts based on their phytochemical profiles and amounts of target bioactive compounds. Chemical composition varied among the analyzed willow species and also among the plant organs of the same species. The major bioactive compounds of the extracts were salicin, chlorogenic acid, rutin and epicatechin. The extracts exhibited significant DPPH^●^ and ^●^OH scavenging activities. Results of molecular docking revealed that chlorogenic acid had the highest binding affinity toward TNF-α and IL-6. UAE extracts represent valuable sources of antioxidant and anti-inflammatory compounds.

## 1. Introduction

Rheumatoid arthritis (RA) is a chronic inflammatory disorder characterized by pain, swelling and destruction of synovial joints [1]. Pro-inflammatory cytokines, such as tumor necrosis factor alpha (TNF-α) and interleukin-6 (IL-6), are crucial in the initiation and progression of inflammation associated with RA. Inhibitors of these cytokines have been proposed as possible anti-inflammatory drug candidates [2]. Furthermore, free radicals play a significant role in the inflammatory process and in the pathogenesis of RA [3]. The imbalance between free radical production and antioxidant defenses, known as oxidative stress, is thought to contribute to inflammatory diseases [4]. Oxidative stress is one of the key factors involved in the pathogenesis of RA as it can activate different signal transduction pathways involved in the expression of pro-inflammatory cytokines and also occur through DNA, lipid and protein damage, resulting in synovial inflammation [5].

Herbal medicines and their active constituents and dietary bioactive compounds that exhibit antioxidant and anti-inflammatory properties are regarded as possible options for treating RA by regulating reactive oxygen species levels [6,7]. Willow bark (*Salix* spp., Salicaceae) is a traditional herbal remedy used to treat pain, fever and inflammatory conditions. Willow leaves, mainly treated as waste in the past, are nowadays recognized as valuable sources of bioactive compounds [8]. Although pharmacological effects of willow bark are primarily attributed to its salicin content, many studies have demonstrated that other constituents play a role as well [9,10,11,12]. Khayyal et al. (2005) studied the mechanism involved in the anti-inflammatory effect of a standardized willow bark extract. The extract was found to be more effective than acetylsalicylic acid in suppressing the rise in TNF-α, IL-1 and IL-6 levels, suggesting that constituents other than salicin contribute to the overall activity [3]. Besides salicin, flavonoids and phenolic acids represent some of the major classes of bioactive compounds in willow bark and leaves [13,14]. Phenolic compounds exhibit a wide range of biological effects; most importantly, the ability to inactivate free radicals deriving from oxidative stress. Phenolic compounds have been reported as the major contributors to the antioxidant activity of plant extracts [15,16]. The anti-inflammatory effect of some phenolic compounds has been found to be closely related to their antioxidant activity [17]. Earlier studies have shown that various *Salix* species exhibit strong antioxidant activity [8,13,18].

In the quest for novel therapeutic agents from medicinal plants, the choice of the extraction solvent and technique is a crucial step that determines the phytochemical profile of the extracts and, consequently, their bioactivity [19]. Nowadays, as environmental awareness is rising, there is a global trend towards replacing conventional extraction techniques (i.e., maceration, Soxhlet extraction) with environmentally friendly, green extraction techniques, such as microwave- and ultrasound-assisted extraction (MAE and UAE). UAE has been widely applied to improve the extraction efficiency for compounds from various sources [20]. Our previous studies revealed the chemical and biological activity profiles of bark and leaf extracts of various species of the genus *Salix* obtained by maceration [13] and MAE [8]. In another paper, the chemical composition and antioxidant activity of *S. eleagnos* bark extracts obtained by maceration, MAE and UAE were analyzed [21]. However, a comparative study of the biological activities and chemical composition of bark and leaf extracts of different species of the genus *Salix* obtained by UAE has not been carried out until now. Therefore, this study aimed to explore the chemical profiles and antioxidant activity of ultrasonic bark and leaf extracts of six willow species: *S. alba*, *S. amplexicaulis*, *S. babylonica*, *S. fragilis*, *S. purpurea* and *S. triandra*. In addition, the anti-inflammatory activity of bioactive compounds found in willow extracts was investigated using in silico molecular docking through assessment of their inhibitory potential against TNF-α and IL-6.

## 2. Results

### 2.1. Chemical Characterization of Extracts

Preliminary chemical characterization included determination of the extraction yield and the total phenolic and flavonoid content of willow bark and leaf extracts obtained using ultrasound-assisted extraction (UAE) (Table 1). Extraction yields of the analyzed *Salix* species were in the ranges of 16.12–24.76% and 27.20–33.25% for bark and leaf samples, respectively. The highest extraction yields for both plant organs were obtained for *S. amplexicaulis*.

The total phenolic content in the examined extracts varied from 4.94 to 50.86 mg gallic acid equivalents per g of dry extract (GAE/g d.e.) for bark and 1.92 to 26.96 mg GAE/g d.e. for leaf extracts (Table 1). The highest concentration of total phenolics in both plant organs was measured in *S. purpurea*. The content of total flavonoids ranged from 1.80 to 17.48 mg quercetin equivalents per g of dry extract (QE/g d.e.) in bark and 1.48 to 29.25 mg QE/g d.e. in leaf samples. The highest flavonoid concentration was found in leaves of *S. amplexicaulis*. Among the bark samples, *S. purpurea* had the highest level of total flavonoids.

**Figure 1:**
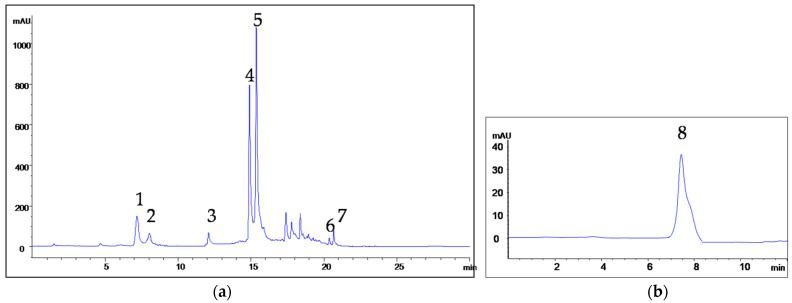
Chromatograms of *S. amplexicaulis* bark extract: (**a**) 1—chlorogenic acid, 2—*p*-hydroxybenzoic acid, 3—epicatechin, 4—*p*-coumaric acid, 5—rutin, 6—quercetin, 7—naringenin; (**b**) 8—salicin.

A more detailed phenolic and flavonoid profiling of bark and leaf samples of the examined *Salix* species revealed variations in the chemical composition and concentrations of target compounds (Table 2 and Table 3). Identification of bioactive compounds was performed using HPLC-DAD by comparing their retention times and UV spectra with those of the standards. The following compounds were identified and quantified: the salicylic glycoside salicin; phenolic acids—gallic, chlorogenic, *p*-hydroxybenzoic (PHB), syringic, *p*-coumaric and *trans*-cinnamic acids; and flavonoids—epicatechin, rutin, quercetin and naringenin. A representative chromatogram of *S. amplexicaulis* bark extract is presented in Figure 1. The main abundant components in the analyzed samples were salicin, chlorogenic acid and rutin.

Salicin was the dominant component in 11 out of 12 samples. It was not detected in leaves of *S. alba*. Salicin concentration ranged from 2.7 to 21.50 mg/g, being the highest in the leaves of *S. amplexicaulis*. High amounts of salicin were also found in leaves of *S. purpurea*, as well as bark of *S. purpurea* and *S. amplexicaulis*. Notably, chlorogenic acid, p-coumaric acid, epicatechin, rutin, quercetin and naringenin were found in bark and leaves of all analyzed *Salix* species, but their concentrations varied among samples. In contrast, gallic, PHB and syringic acids were detected only in half of the examined samples. Gallic acid was found in both plant organs in *S. babylonica*, *S. fragilis* and *S. triandra*. PHB was present in bark and leaves of *S. amplexicaulis* and *S. babylonica*, but only in bark of *S. alba* and *S. fragilis*. Syringic acid was detected in low amounts in bark and leaves of *S. alba*, *S. babylonica* and *S. triandra*. *p*-Coumaric acid levels were significantly higher in *S. amplexicaulis* and *S. purpurea* in comparison to the other species. *trans*-Cinnamic acid was not detected in *S. amplexicaulis*, while it was measured in all the other analyzed willow species. In terms of trans-cinnamic acid content, the leaves and bark of *S. fragilis* stood out as samples with high concentrations of this phenolic acid.

Among the flavonoid compounds, rutin was the dominant one in most of the samples, except bark of *S. babylonica*, *S. fragilis* and *S. triandra* and leaves of *S. fragilis*. In the latter four samples, epictechin was the dominant flavonoid. The highest concentration of epicatechin was determined in leaves of *S. fragilis*, while the lowest was in bark of *S. alba*. The level of rutin was the highest in leaves of *S. amplexicaulis*, followed by leaves of *S. purpurea*. High amounts of rutin were also found in the bark of these two willow species. The difference in rutin content between the leaves and bark of the same *Salix* species was evident in most species, being 2–2.7-fold higher in leaves than bark, with the exception of *S. alba* and *S. babylonica*, where the rutin level was slightly higher in bark. With respect to quercetin concentrations, the highest was measured in leaves of *S. amplexicaulis*. Higher levels of quercetin were found in the leaves than the bark in half of the analyzed *Salix* species (namely, *S. amplexicaulis* (2.7-fold), *S. fragilis* (2.5-fold) and *S. purpurea* (1.5-fold)), while in the other three willow species, there were no significant differences in quercetin amounts between the two plant organs. Naringenin concentration was the highest in leaves of *S. purpurea*, followed by leaves of *S. amplexicaulis*. Bark of *S. purpurea* was the richest in naringenin among the bark samples of the analyzed *Salix* species.

### 2.2. Chemometric Analysis

**Figure 2:**
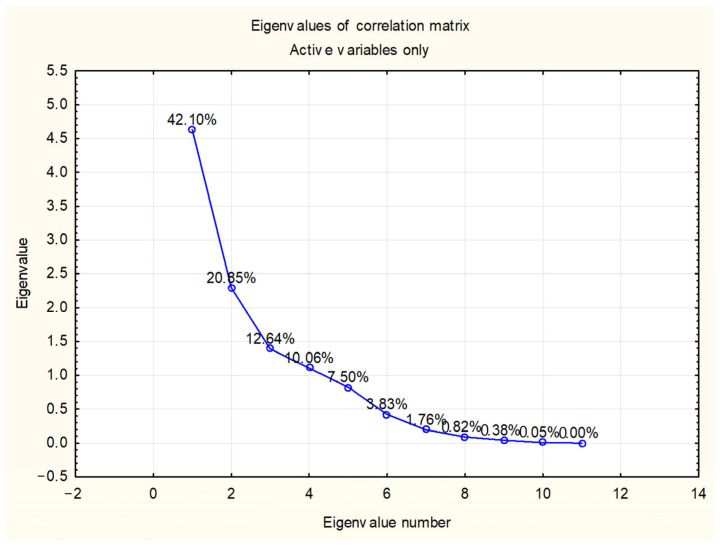
Eigenvalues of correlation matrix.

Principal component analysis (PCA) was performed to discriminate and classify *Salix* species according to their chemical composition and the content of the target compounds. Principal components (PCs) were obtained by decomposing the original data matrix into loading and score vectors. The PC accounting for most of the variation in the dataset was the first PC (PC1), the one accounting for the next largest proportion of variation was the second (PC2) and so forth. PC1 and PC2 covered 62.96% of the total variance, while the first four PCs explained cumulatively 85.65% of the total variance (Figure 2).

**Figure 3:**
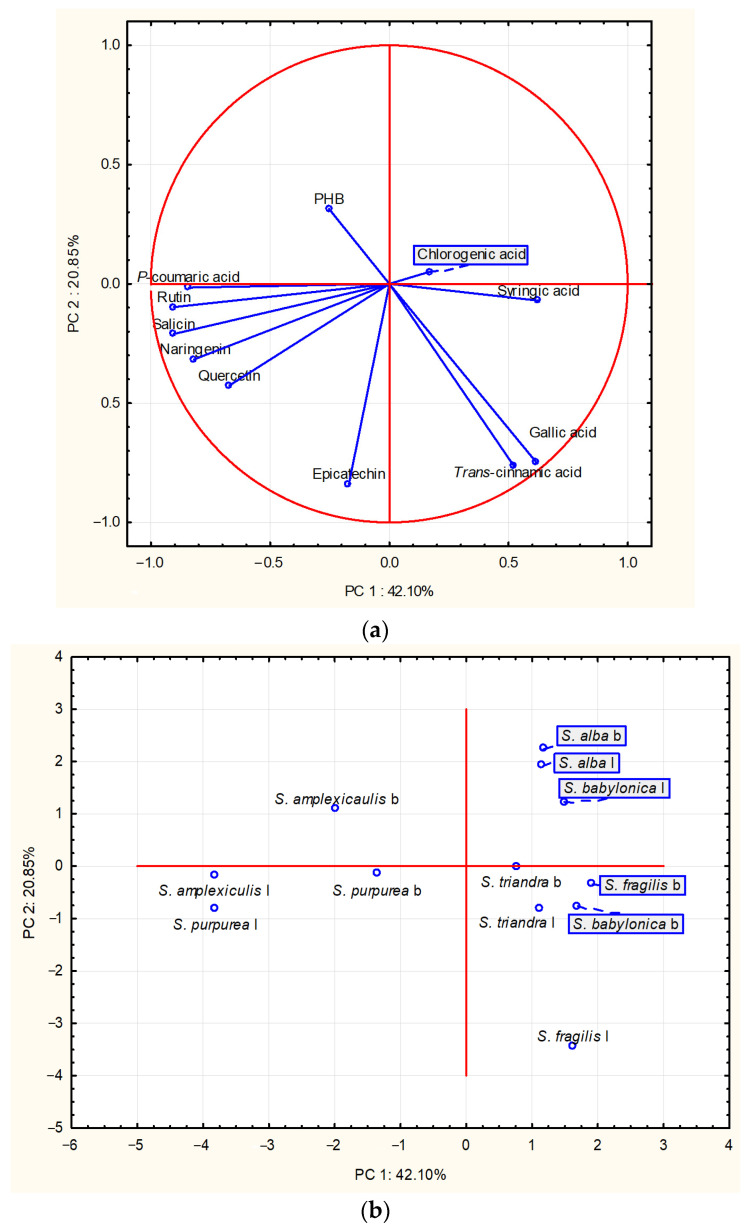
Principal component analysis. Projection of the examined (**a**) variables—target compounds—and (**b**) cases—samples in the space defined by the first two principal components. PHB—*p*-hydroxybenzoic acid; b—bark; l—leaf.

The results of the PCA are presented in Figure 3. The variability described by PC1 mostly correlated with salicin, rutin, p-coumaric acid and naringenin concentrations, while PC2 was correlated with those of epicatechin, trans-cinnamic and gallic acid. PC1 allowed the differentiation of two groups according to similarity in the chemical profiles of the analyzed samples. Bark and leaf samples of *S. amplexicaulis* and *S. purpurea* comprised the first group, which was characterized by high salicin, rutin and p-coumaric acid levels. The bark of *S. amplexicaulis* was further separated as a result of p-hydroxybenzoic acid content, which was the highest among the analyzed samples. The second group consisted of the bark and leaf samples of the other four *Salix* species. These samples were clustered into three subgroups, one consisting of *S. alba* bark and leaves and *S. babylonica* leaves, the second of *S. babylonica* and *S. fragilis* bark and *S. triandra* bark and leaves and the third only of *S. fragilis* leaves. The content of trans-cinnamic acid allowed discrimination of *S. fragilis* leaves from the other samples.

### 2.3. Antioxidant Activity

The antioxidant potential of the bark and leaf extracts of *Salix* species obtained with UAE was evaluated using DPPH^•^ and ^•^OH scavenging assays. Concentrations of extracts that inhibited 50% (IC_50_) DPPH^•^ were in the range from 3.32 to 44.31 μg/mL, the lowest being in the bark extract of *S. alba*, implying that this sample had the highest antiradical capacity. The lowest DPPH^•^ scavenging activity was demonstrated for *S. babylonica* leaf extract (Table 4). It was also observed that bark extracts had stronger DPPH^•^ quenching abilities than leaf extracts of the same *Salix* species, except for *S. fragilis*, where the opposite applied.

The examined bark and leaf samples of the *Salix* species also showed notable potential to quench ^•^OH. IC_50_ values were within the range of 15.35 to 36.28 μg/mL (Table 4). The ^•^OH scavenging ability of the *S. alba* leaf extract was the most pronounced, while that of the bark extract of *S. babylonica* was the least pronounced. Comparing the ^•^OH scavenging capacities of bark and leaf extracts of the same willow species, it can be noticed that leaf extracts of *S. alba*, *S. amplexicaulis* and *S. babylonica* showed greater ^•^OH scavenging effects than bark extracts of these species, while bark extracts of *S. fragilis*, *S. purpurea* and *S. triandra* quenched ^•^OH more effectively than the leaf extracts.

It can also be noted that the radical scavenging ability of the examined extracts was stronger against DPPH^•^ than ^•^OH, with the exception of *S. babylonica* bark extract.

### 2.4. Molecular Docking

A molecular docking study was conducted to investigate the interaction between bioactive compounds of willow extracts and pro-inflammatory cytokines TNF-α and IL-6 in order to assess the anti-inflammatory potential of these compounds. The conformation with the lowest binding energy for each ligand was considered as the most favorable. The results of molecular docking analysis of *Salix*-derived compounds against the target proteins are summarized in Table 5 and Table 6. A well-known anti-inflammatory drug, acetylsalicylic acid, was used as control.

**Figure 4:**
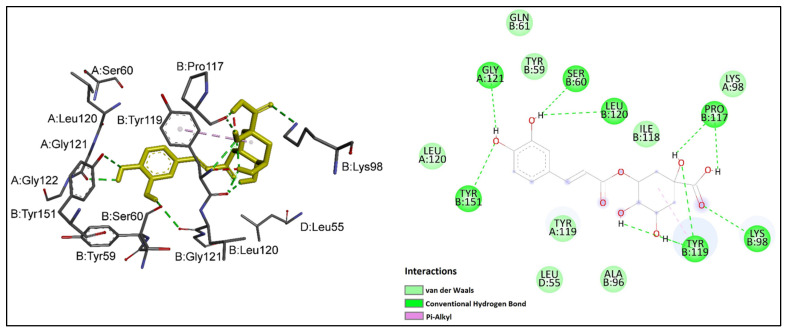
Chlorogenic acid (shown in yellow) interactions at the active site of TNF-α. Left: three-dimensional display; right: two-dimensional display.

Chlorogenic acid showed the greatest inhibitory potential against TNF-α among the studied ligands with a binding affinity of −7.55 kcal/mol, followed by naringenin (−6.93 kcal/mol). The flavonoid compounds epicatechin and quercetin demonstrated similarly high binding affinities toward TNF-α. According to the binding energy values presented in Table 5, the binding affinities of these four ligands were higher than that of the positive control acetylsalicylic acid. Rutin, the rest of the phenolic acids and salicin had lower binding affinities than acetylsalicylic acid. Among the studied ligands, *trans*-cinnamic acid showed the weakest binding affinity toward TNF-α. Chlorogenic acid, in addition to having the lowest binding energy, also interacted with the largest number of crucial residues at the active site of TNF-α. The binding mode interactions of chlorogenic acid and naringenin, as ligands with notable inhibitory potential against TNF-α, were explored in more detail. Interactions of chlorogenic acid with TNF-α are shown in Figure 4. It could be observed that the carboxyl and hydroxyl groups of chlorogenic acid established nine hydrogen bonds with the side chains of important residues at the interaction site of TNF-α; namely, SerB 60, LysB 98, ProB 117, TyrB 119, LeuB 120, GlyA 121 and TyrB 151. An additional pi–alkyl interaction was observed between chlorogenic acid and Tyr B 119. Moreover, the hydrophobic cleft formed by TyrB 59, GlnB 61, AlaB 96, LysA 98, IleB 118, TyrA 119 and LeuA 120 provided additional stability to the ligand in the active site of TNF-α.

**Figure 5:**
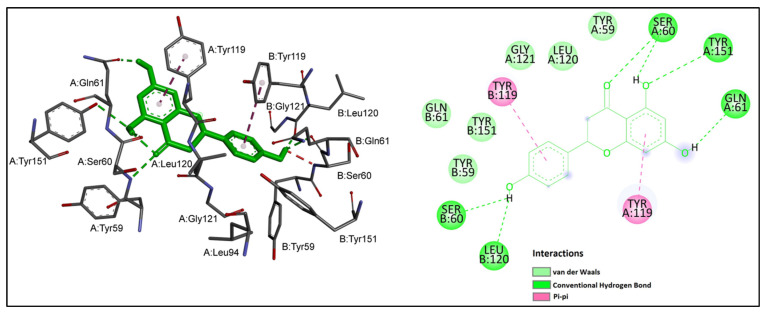
Naringenin (shown in green) interactions at the active site of TNF-α. Left: three-dimensional display; right: two-dimensional display.

The detailed interaction pattern of naringenin is shown in Figure 5.

It could be observed that naringenin formed hydrogen bonds with SerA 60, SerB 60, GlnA 61, LeuB 120 and TyrA 151, while van der Waals interactions were formed with TyrA 59, TyrB 59, GlnB 61, LeuA 120, Gly A 121 and TyrB 151. The benzene rings of naringenin established pi–pi stacking interactions with the aromatic rings of TyrA 119 and TyrB 119. Tyr 119 is reported to be crucial for TNF-α inhibition [22].

With respect to the inhibitory potential of *Salix*-derived bioactive compounds toward IL-6, chlorogenic acid once again showed the greatest binding affinity, followed by p-coumaric acid and quercetin (Table 6). Rutin, salicin and syringic acid were weak inhibitors of IL-6, with binding energies higher than that of acetylsalycilic acid.

Analysis of protein–ligand interactions showed that most of the ligands could interact with residues Leu 178, Arg 179 and Arg 182. Key residues at the active site of IL-6 included Phe 74, Phe 78, Leu 178, Arg 179 and Arg 182 [23].

**Figure 6:**
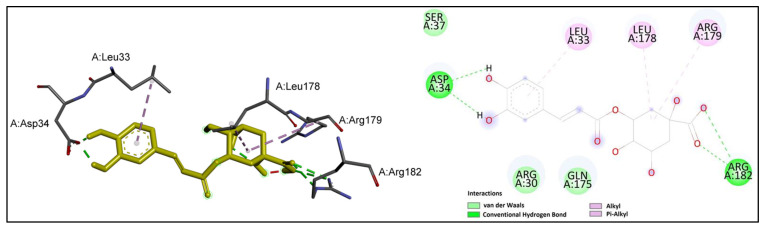
Chlorogenic acid (shown in yellow) interactions at the active site of IL-6. Left: three-dimensional display; right: two-dimensional display.

The interactions of chlorogenic acid at the active site of IL-6 are shown in Figure 6.

It could be observed that the carboxyl group of chlorogenic acid established hydrogen bonds with the key residue Arg 182, while the hydoxyl groups on the aromatic ring formed two hydrogen bonds with Asp 34. Moreover, the prominent residues Leu 178 and Arg 179 were involved in alkyl interactions with chlorogenic acid.

**Figure 7:**
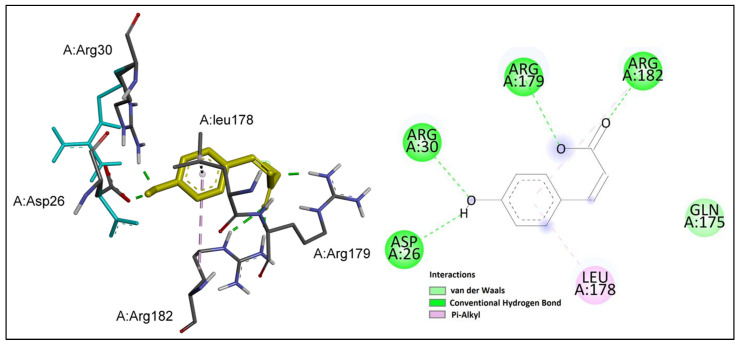
*p*-Coumaric acid (shown in yellow) interactions at the active site of IL-6. Left: three-dimensional display; right: two-dimensional display.

*p*-Coumaric acid showed good inhibitory potential against IL-6, with the binding affinity similar to that of chlorogenic acid. The binding-mode interactions of *p*-coumaric acid are shown in Figure 7.

*p*-Coumaric acid formed several hydrogen bonds with the key residues at the active site of IL-6; namely, Arg 179 and Arg 182. In addition, the phenol group was involved in hydrogen bond interactions with residues Asp 26 and Arg 30. Moreover, pi–alkyl interactions were observed between the benzene ring and key residues Leu 178 and Arg 182.

## 3. Discussion

The discovery of bioactive compounds from natural sources with a wide range of applications and potential health benefits is in the spotlight of much research. The quest for new sources and more efficient and selective extraction techniques is underway. In this study, a comparative assessment of the phytochemical composition and antioxidant effects of extracts obtained via UAE of two plant organs (bark and leaves) of various species of the genus *Salix* was revealed for the first time. UAE is a technique that employs ultrasound waves to intensify the extraction process via mechanical, cavitation and thermal effects, leading to cell wall disruption, particle size reduction and enhanced mass transfer [24]. These features make UAE suitable for the extraction of phenolic compounds from plant matrices [25]. There has been a growing trend to replace conventional extraction techniques commonly used for isolation of bioactive compounds from medicinal plants (maceration, Soxhlet extraction) with alternative ones that are more efficient, faster, sustainable, energy-saving and exempt from toxic organic solvents. UAE is an example of such an environmentally friendly, “green” extraction technique [26]. Water and ultrasonication have been found to be an effective extraction solvent and technique for the extraction of phenolic compounds and flavonoids [27]. In this study, the extraction yields achieved with UAE (solvent: water; extraction time: 30 min) for bark and leaf samples of willow species were significantly higher than those obtained with maceration of the same species with 70% (*v*/*v*) ethanol (extraction time: 48 h) [13]. Detailed chemical characterization of the extracts revealed that salicin, chlorogenic acid, rutin and epicatechin were the major bioactive compounds extracted from the bark and leaves of various *Salix* species, which is in agreement with previous reports [8,13,18,28,29]. Salicin, in synergy with phenolic acids and flavonoids, contributes to the analgesic and anti-inflammatory effects of willow extracts [9,12]. Samples with outstanding salicin amounts were *S. amplexicaulis* and *S. purpurea;* particularly, in the leaves of these species. The bark of *S. purpurea* is regarded as one of the richest salicin-containing organs, with well-documented medicinal use by the European Medicine Agency (2017) [30]. In addition, the leaves of *S. amplexicaulis*, a lesser-known species from the same subsection Purpurea (subgenus Vetrix, section Helix) [31], are emerging as novel sources of this bioactive compound. Chlorogenic acid is one of the dominant phenolic compounds in the human diet, found in coffee, fruits and vegetables, and shows antioxidant, anti-inflammatory, neuroprotective, antiviral and anticancer activities [32]. Therefore, consumption of chlorogenic acid-rich products is linked with diverse potential health benefits. Chlorogenic acid was found in significant quantities in the analyzed willow samples, especially in the bark of *S. fragilis* and *S. triandra*, and it was comparable or even higher than the chlorogenic acid content reported for fruits and vegetables [33]. Rutin and epicatechin were the dominant flavonoid compounds in the analyzed willow samples, which is consistent with other studies [18,29]. The better efficiency of UAE in comparison to maceration with ethanol was particularly evident for the extraction of salicin, as its levels in the ultrasonic extracts of all analyzed willow samples were significantly higher than those in ethanol extracts of the same species. Also, higher amounts of chlorogenic acid in ultrasonic extracts in comparison to ethanol extracts were achieved in 7 out of 12 willow samples (bark of *S. alba*, bark and leaves of *S. amplexicaulis*, leaves of *S. babylonica* and bark of *S. fragilis*, *S. purpurea* and *S. triandra*). However, rutin content was higher than that of ethanol extracts in only two ultrasonic extracts (leaves of *S. fragilis* and *S. triandra*) and epicatechin content was higher in four ultrasonic extracts (bark and leaves of *S. amplexicaulis* and leaves of *S. fragilis* and *S. triandra*) [13].

In addition, a chemometric tool—principal component analysis—was applied to differentiate the sample extracts based on their phytochemical profiles and amounts of the target bioactive compounds. This may give a direction for the selection of extracts with the most favorable composition that could be further utilized for various applications in the pharmaceutical industry.

The growing interest in research on natural products derives mainly from their antioxidant effects. Antioxidants can reduce the incidence of oxidative stress-related diseases by neutralizing free radicals and reactive species, thereby counteracting oxidative damage and interrupting free radical-mediated chain reactions. In this study, the ability of the ultrasonic bark and leaf extracts of various *Salix* species to scavenge DPPH^•^ and ^•^OH was assessed. The DPPH assay is one of the most widely used methods for screening the antioxidant potential of plant extracts. It is suitable for the assessment of both lipophilic and hydrophilic compounds, regardless of the nature of the antioxidants [34]. ^•^OH is one of the most potent and reactive natural free radicals, directly involved in the irreversible damage caused by oxidative stress and contributing to many human diseases, such as inflammatory, malignant, cardiovascular and neurodegenerative diseases [34,35]. The analyzed ultrasonic extracts of *Salix* species demonstrated significant radical scavenging capacities. According to the antioxidant assay results, radical scavenging ability varied among the analyzed willow species and also between the two plant organs within the same species. In comparison to the antioxidant activity of extracts obtained using microwave-assisted extraction of these species, the DPPH^●^ scavenging ability of the ultrasonic extracts was weaker for most samples but stronger for the leaf extract of *S. amplexicaulis,* as well as the bark extracts of *S. purpurea* and *S. triandra* [8]. Also, most of the ultrasonic extracts displayed lower DPPH^●^ scavenging potential when compared to that of the ethanolic extracts of the same willow species, but it was higher in the case of *S. triandra* bark extract [13]. In contrast, the antioxidant activities of the majority of the ultrasonic extracts of the willow samples in the ^●^OH assay were higher than those of microwave extracts [8]. Furthermore, the ultrasonic extracts of the analyzed *Salix* species were more potent ^●^OH quenchers than ethanolic extracts of the same species [13].

There is a close relationship between the mediators of inflammation and free oxygen radicals. Inflammatory stimuli are known to trigger the release of reactive oxygen species, such as superoxide anion, hydrogen peroxide, ^●^OH and singlet oxygen [1]. Inflammation is activated by pro-inflammatory mediators, such as the cytokines TNF-α and IL-6. Cytokines are the major signaling molecules released by inflammatory cells and are involved in multiple functions. TNF-α is one of the earliest and most important inflammatory mediators, mainly produced by macrophages and mast cells. TNF-α has multiple roles in the inflammatory response: activation of inflammatory cytokines coded by the NF-κB signal pathway, facilitation of adhesion molecules, initiation of gene expression of prostaglandin synthesis pathway enzymes (e.g., cyclooxygenase-2) and induction of nitric oxide synthase, leading to the activation of endothelium and white blood cells [36]. Its dysregulation has been linked with cancer, neurological diseases and autoimmune diseases, including RA [22]. IL-6 participates in the production of reactive species and in the synthesis of inflammatory molecules (chemokines, integrins and matrix metalloproteinases). The major sources of IL-6 are macrophages and T cells [36]. Willow bark is widely accepted as an analgesic and antirheumatic drug. Its anti-inflammatory action has been demonstrated in experimental studies with different models of inflammation: carrageenan rat paw edema, adjuvant-induced arthritis models, chondrocytes, LPS-activated human monocytes and differentiated macrophages and THP-1-derived human macrophages [10]. Although salicin was first suggested to be responsible for the anti-inflammatory effects of willow bark, its contribution was found to be less prominent, while fractions of polyphenols, flavonoids and proanthocyanidins were essential for the effect of willow bark extracts [10,12]. However, the contributions of individual compounds have not been determined. In this study, molecular docking analysis was conducted to explore the ability of bioactive compounds extracted from willow species to act as inhibitors of pro-inflammatory cytokines TNF-α and IL-6. The most common values for the selection of potential candidates currently accepted in drug design are values less than −6.0 kcal/mol for binding free energy. However, there is still no consensus on the range that binding energies should fall within for biologically active compounds [37]. Having binding energies below the commonly accepted value (−6.0 kcal/mol), chlorogenic acid, naringenin, epicatechin and quercetin demonstrated significant inhibitory potential against TNF-α, while chlorogenic acid, *p*-coumaric acid and quercetin demonstrated significant inhibitory potential against IL-6. Among the studied ligands, chlorogenic acid had the strongest binding affinity toward TNF-α and IL-6, suggesting its important role in the anti-inflammatory activity of willow extracts. The low binding affinity of salicin toward TNF-α and IL-6 is in line with previous reports [3,10,12]. Chlorogenic acid, naringenin, epicatechin and quercetin displayed greater inhibition of TNF-α in comparison to the control inhibitor acetylsalicylic acid (−5.83 kcal/mol). In contrast, most of the docked ligands had lower binding energies and greater affinity toward IL-6 compared to acetylsalicylic acid (−4.66 kcal/mol), with the exception of syringic acid, salicin and rutin, which were found to be weak inhibitors of IL-6. The stronger inhibitory effect of acetylsalicylic acid on TNF-α than IL-6 is consistent with experimental data [38].

## 4. Materials and Methods

### 4.1. Reagents and Standards

2,2-Diphenyl-1-pycrylhydrazil (DPPH) was obtained from Alfa Aesar (Karlsruhe, Germany) and acetonitrile, methanol, orthophosphoric acid, tetrahydrofuran and acetic acid from J.T. Baker (Deventer, The Netherlands). Chlorogenic acid (≥95%), *p*-hydroxybenzoic acid (≥99%), syringic acid (≥95%), rutin (≥94%), naringenin (≥98%), epicatechin (≥98%) and *trans*-cinnamic acid (≥99%) were purchased from Sigma-Aldrich (St. Louis, MI, USA); *p*-coumaric acid (≥98%) from Fluka (Buchs, Switzerland); quercetin (≥99%) from Extrasynthese (Genay, France); and salicin (>90%) from Carl Roth GmbH (Karlsruhe, Germany). All other reagents were of analytical grade.

### 4.2. Plant Material

Collection sites and dates for the bark and leaves of the six species of the genus *Salix* L. were as follows: *S. alba* L. 1753—Pecenjevce (43°06′01″ N, 21°54′60″ E), June 2014; *S. amplexiaculis* Bory et Chaub. 1838—Pecenjevce (43°06′01″ N, 21°54′60″ E), June 2014; *S. babylonica* L. 1753—Bosut riverside, Morovic (44°59′31″ N, 19°12′22″ E), September 2013; *S. fragilis* L. 1753—Vrdnik (45°07′13″ N, 19°47′16″ E), June 2013; *S. purpurea* L. 1753 subsp. *purpurea*—Mountain Deli Jovan (44°02′11.7″ N, 22°12′49.49″ E), August 2013; *S. triandra* L. 1753—Vlasina Lake (42°42′26″ N, 22°20′32″ E), July 2013. Voucher specimens were confirmed and deposited in the Herbarium of the University of Novi Sad, Faculty of Sciences, Department of Biology and Ecology—Herbarium BUNS (nos 2-1471–2-1474 and 2-1477–2-1482). The plant material was air-dried and stored at room temperature until the time of analysis. Dried willow bark and leaves were ground in an electric mill (Bosch, Gerlingen, Germany) and particle size diameter (d = 0.35 mm) was determined with a sieve set (Retsch GmbH and Co., KG, Haan, Germany).

### 4.3. Extraction Procedure

Ultrasound-assisted extraction (UAE) was carried out in an ultrasonic bath (Labsonic Falc, Treviglio, Italy) with an ultrasound frequency of 40 kHz, temperature of 25 °C and extraction time of 30 min [21]. In each experiment, 0.5 g of grounded sample was mixed with 50 mL of distilled water in a 200 mL Erlenmeyer flask and placed in the ultrasonic bath. After extraction, the extracts were filtered, evaporated to dryness under a vacuum and left to dry in a desiccator for 24 h. The dry extract (d.e.) was weighed and extraction yields were calculated (%, g of d.e./100 g of sample). The obtained d.e. was dissolved in 95% (*v*/*v*) methanol for further analysis.

### 4.4. Total Phenolic and Flavonoid Content Determination

Quantification of the total phenolics and flavonoids was performed spectrophotometrically (Agilent 8453 117 UV-Visible Spectroscopy System, Santa Clara, CA, USA) using Folin–Ciocalteu and aluminum chloride colorimetric assays, respectively [13]. The concentration of total phenolics was expressed as mg of gallic acid equivalents (GAE) per g of d.e. (mg GAE/g d.e.) using a standard curve of gallic acid and that of total flavonoids as mg of quercetin equivalents (QE) per g of d.e. (mg QE/g d.e.) using a standard curve of quercetin. All measurements were performed in triplicate.

### 4.5. High-Performance Liquid Chromatography (HPLC) Analysis

Detailed chemical characterization of extracts was performed with two HPLC methods (one for determination of salicin, the other for phenolic acids and flavonoids) using an Agilent HP 1100 HPLC diode array detection (DAD) system equipped with an autosampler (Santa Clara, CA, USA) [8]. Salicin content was determined with a method based on the previous report by Guvenc et al. (2007) [39] using a Zorbax CB-C18 column (4.6 × 150 mm, 5 μm particle size) and a mobile phase consisting of bidistilled water, tetrahydrofuran and *ortho*-phosphoric acid (97.7:1.8:0.5) (*v*/*v*/*v*) delivered in isocratic mode. Determination of gallic, chlorogenic, *p*-hydroxybenzoic, syringic, *p*-coumaric and *trans*-cinnamic acids and epicatechin, rutin, quercetin and naringenin was performed using a Zorbax CB-C18 column (4.6 mm × 150 mm, 5 μm); mobile phase—solvent A: 0.1% acetic acid in deionized water, solvent B: 0.1% acetic acid in acetonitrile delivered in gradient mode—3.25 min—10% B, 8 min—12% B, 15 min—25% B, 15.8 min—30% B, 25 min—90% B, 25.4 min—100% B. The injection volume was 10 μL, the flow rate was 1 mL/min, UV detection took place at 280 nm and the duration of analysis was 30 min. Both methods were validated using standards of the examined compounds prior to the injection of the extracts and a calibration curve for each standard was constructed. The amounts of the quantified compounds were expressed as mg/g of dry plant material.

### 4.6. Chemometric Analysis

Principal component analysis (PCA) was performed with Statistica v. 12 software (Stat Soft Inc., Tulsa, OK, USA). The samples (bark and leaves of different *Salix* species) represented the cases, while the detected amounts of bioactive compounds were the variables. All data were standardized prior to calculation.

### 4.7. Antioxidant Activity

The radical scavenging activity (DPPH^●^ and ^●^OH) of the *Salix* bark and leaf extracts was measured using spectrophotometric methods described previously [21].

### 4.8. Molecular Docking Analysis

Chemical structures of ligand molecules (salicin, gallic acid, chlorogenic acid, *p*-hydroxybenzoic acid, syringic acid, *p*-coumaric acid, *trans*-cinnamic acid, epicatechin, rutin, quercetin and naringenin) were taken from the PubChem database (http://pubchem.ncbi.nlm.nih.gov/ accessed on 1 June 2023). The structures of molecules were geometrically optimized using the software Avogadro 2.0 following the MMFF94 method [40].

The three-dimensional crystal structures of TNF-α (pdb code: 2az5) [41] and IL-6 (pdb code: 1alu) [42] were retrieved from the Protein Data Bank (PDB) (http://www.rcsb.org/ accessed od 1 June 2023). The target proteins were prepared by removing all water molecules, heteroatoms, any co-crystallized solvent and ligands before docking the ligands of interest (compounds found in *Salix* samples). AutoDockTools (ADT; version 1.5.6) was used to add polar hydrogen atoms, merge non-polar hydrogen atoms and apply Gasteiger charges [43]. The grid box was designed to include the entire binding site of the target proteins centered on the cognate ligand (x = −19.4096, y = 74.6508, z = 33.8496 and x = 7.5087, y = −12.8277, z = 0.056500 for TNF-α and IL-6, respectively). The dimensions of the grid box were 60 × 60 × 60 with a distance of 0.375 Å between points. Molecular docking was conducted using the AutoDock 4.2.3 program package (Molecular Graphics Laboratory, La Jolla, CA, USA). Docking simulations were performed using the Lamarckian Genetic Algorithm [43] with a standard docking procedure for rigid receptors and flexible ligands. A total of 25 runs, along with 25 × 10^5^ energy evaluations and 27,000 iterations, were carried out. Other parameters were set to default. Conformations of docked structures with the lowest binding energies were considered as the most favorable docking poses. Discovery Studio Visualizer 4.5 (DSV; Dassault Systèmes BIOVIA, San Diego, CA, USA) was used to visualize binding interactions and produce the figures. Docking procedures were validated by re-docking the co-crystallized ligand. An RMSD value for the re-docked conformation and the original structure less than 2 Å indicated the reliability of the binding ability prediction for new ligands.

### 4.9. Statistical Analysis

Statistical analyses were performed using IBM SPSS, version 22. The data are reported as mean values ± standard deviation (SD). Mean values of the measured parameters were subjected to one-way analysis of variance (ANOVA) using Duncan’s multiple range test to determine significant differences among samples with a level of significance *p* < 0.05.

## 5. Conclusions

A comparative assessment of the phytochemical composition and antioxidant effects of extracts obtained through ultrasound-assisted extraction of two plant organs (bark and leaves) of various species of the genus *Salix* was revealed for the first time in this study. The major bioactive compounds of the extracts were salicin, chlorogenic acid, rutin and epicatechin. Principal component analysis was applied to differentiate the sample extracts based on their phytochemical profiles and amounts of target bioactive compounds. Extracts of *S. amplexicaulis* and *S. purpurea* showed favorable chemical profiles with high contents of the target bioactive compounds and could be further utilized for various applications in the pharmaceutical industry. The analyzed willow extracts showed significant antioxidant activity. In addition, molecular docking studies demonstrated the anti-inflammatory potential of *Salix*-derived compounds targeting TNF-α and IL-6. Chlorogenic acid exhibited the strongest binding affinity towards both cytokines. This research represents a step forward in the understanding of the anti-inflammatory effects of willow extracts that could pave the path for the development of new therapeutics in the treatment of inflammatory disorders, such as rheumatoid arthritis.

## Figures and Tables

**Table 1:** Extraction yield and total phenolic and total flavonoid content in bark and leaf extracts of *Salix* species.

Sample	Extraction Yield (%)		Total Phenolics (mg GAE/g d.e.)		Total Flavonoids (mg QE/g d.e.)	
	Bark	Leaf	Bark	Leaf	Bark	Leaf
*S. alba*	20.74 ± 0.67 ^a^	30.21 ± 0.75 ^b^	25.14 ± 0.90 ^a^	1.92 ± 0.24 ^b^	1.8 ± 0.20 ^a^	6.25 ± 0.75 ^b^
*S. amplexicaulis*	24.76 ± 0.75 ^c^	33.25 ± 0.80 ^d^	17.15 ± 1.05 ^c^	17.02 ± 1.00 ^c^	15.50 ± 0.50 ^c^	29.25 ± 0.75 ^d^
*S. babylonica*	18.86 ± 0.81 ^e^	29.87 ± 0.87 ^b^	13.23 ± 1.09 ^d^	1.76 ± 0.32 ^b^	1.95 ± 0.33 ^a^	1.48 ± 0.50 ^a^
*S. fragilis*	18.30 ± 0.79 ^e^	27.87 ± 0.84 ^f^	4.94 ± 0.89 ^e^	7.18 ± 0.74 ^f^	1.94 ± 0.36 ^a^	3.52 ± 0.50 ^e^
*S. purpurea*	19.11 ± 0.93 ^e^	32.61 ± 0.57 ^d^	50.86 ± 0.84 ^g^	26.96 ± 0.65 ^h^	17.48 ± 2.17 ^f^	16.66 ± 0.33 ^cf^
*S. triandra*	16.12 ± 0.88 ^g^	27.20 ± 0.81 ^f^	11.75 ± 0.59 ^i^	10.52 ± 0.79 ^i^	7.16 ± 0.77 ^b^	15.09 ± 0.69 ^c^

Results are presented as mean values of triplicate measurements ± SD. Different superscript letters within the same category (yield/total phenolics/total flavonoids) indicate significant differences between means at the 0.05 level. GAE—gallic acid equivalents; d.e.—dry extract; QE—quercetin equivalents.

**Table 2:** Levels of salicin and phenolic acids in bark (b) and leaf (l) extracts.

Species	Salicin	Gallic Acid	Chlorogenic Acid	*p*-Hydroxybenzoic Acid	Syringic Acid	*p*-Coumaric Acid	*trans*-Cinnamic Acid
	mg/g of Dry Plant Material (mg/g of Dry Extract)						
*S. alba* (b)	4.5 ± 0.05 ^a^ (21.70)	n.d.	1.89 ± 0.04 ^a^ (9.11)	0.48 ± 0.00 ^a^ (2.31)	0.19 ± 0.00 ^a^ (0.92)	0.16 ± 0.01 ^ai^ (0.77)	0.03 ± 0.00 ^af^ (0.14)
*S. alba* (l)	n.d.	n.d.	1.93 ± 0.03 ^a^ (6.39)	n.d.	0.06 ± 0.00 ^b^ (0.20)	0.13 ± 0.01 ^b^ (0.43)	0.24 ± 0.29 ^b^ (0.79)
*S. amplexicaulis* (b)	12.20 ± 0.04 ^b^ (49.27)	n.d.	4.20 ± 0.04 ^b^ (16.96)	1.34 ± 0.00 ^b^ (5.41)	n.d.	2.35 ± 0.01 ^c^ (9.49)	n.d.
*S. amplexicaulis* (l)	21.50 ± 0.03 ^c^ (64.66)	n.d.	3.60 ± 0.03 ^c^ (10.83)	0.57 ± 0.01 ^c^ (1.71)	n.d.	0.93 ± 0.01 ^d^ (2.80)	n.d.
*S. babylonica* (b)	7.50 ± 0.04 ^d^ (39.77)	0.59 ± 0.02 ^a^ (3.13)	0.74 ± 0.01 ^d^ (3.93)	0.64 ± 0.01 ^d^ (3.39)	0.30 ± 0.00 ^c^ (1.59)	0.15 ± 0.01 ^a^ (0.80)	0.57 ± 0.01 ^c^ (3.02)
*S. babylonica* (l)	2.7 ± 0.03 ^e^ (9.04)	0.27 ± 0.01 ^b^ (0.90)	2.45 ± 0.01 ^e^ (8.20)	0.21 ± 0.01 ^e^ (0.70)	0.12 ± 0.00 ^d^ (0.40)	0.09 ± 0.00 ^e^ (0.30)	0.09 ± 0.00 ^abf^ (0.30)
*S. fragilis* (b)	7.20 ± 0.06 ^d^ (39.34)	0.47 ± 0.01 ^c^ (2.57)	8.53 ± 0.01 ^f^ (46.61)	0.19 ± 0.01 ^f^ (1.04)	0.13 ± 0.00 ^e^ (0.71)	0.07 ± 0.02 ^f^ (0.38)	1.05 ± 0.02 ^d^ (5.74)
*S. fragilis* (l)	6.80 ± 1.70 ^d^ (24.40)	1.02 ± 0.01 ^d^ (3.66)	1.76 ± 0.01 ^g^ (6.32)	n.d.	0.16 ± 0.00 ^f^ (0.57)	0.28 ± 0.01 ^g^ (1.00)	1.67 ± 0.01 ^e^ (5.99)
*S. purpurea* (b)	12.50 ± 0.03 ^b^ (65.41)	n.d.	1.90 ± 0.01 ^a^ (9.94)	n.d.	n.d.	1.19 ± 0.01 ^h^ (6.23)	0.02 ± 0.00 ^af^ (0.10)
*S. purpurea* (l)	18.50 ± 0.02 ^f^ (56.73)	n.d.	0.79 ± 0.00 ^h^ (2.42)	n.d.	n.d.	2.41 ± 0.02 ^i^ (7.39)	0.03 ± 0.00 ^af^ (0.09)
*S. triandra* (b)	3.90 ± 0.05 ^a^ (24.19)	0.47 ± 0.01 ^c^ (2.92)	8.21 ± 0.01 ^i^ (50.93)	n.d.	n.d.	0.18 ± 0.01 ^aj^ (1.12)	0.17 ± 0.00 ^bf^ (1.05)
*S. triandra* (l)	2.10 ± 0.07 ^e^ (7.72)	0.59 ± 0.01 ^a^ (2.17)	2.73 ± 0.03 ^j^ (10.04)	n.d.	n.d.	0.16 ± 0.01 ^aj^ (0.59)	0.57 ± 0.00 ^c^ (2.10)

Data are presented as means of triplicate measurements ± SD. Different superscript letters within the same column indicate significant differences between means at the 0.05 level. Data in parentheses are presented as means of triplicate measurements. n.d.—not detected.

**Table 3:** Flavonoid content in bark (b) and leaf (l) extracts.

Species	Epicatechin	Rutin	Quercetin	Naringenin
	mg/g of Dry Plant Material (mg/g of Dry Extract)			
*S. alba* (b)	0.35 ± 0.00 ^a^ (1.69)	1.70 ± 0.03 ^a^ (8.20)	0.24 ± 0.18 ^a^ (1.16)	0.33 ± 0.00 ^a^ (1.59)
*S. alba* (l)	0.51 ± 0.00 ^b^ (1.69)	1.60 ± 0.01 ^b^ (5.30)	0.39 ± 0.00 ^abcg^ (1.29)	0.34 ± 0.00 ^b^ (1.13)
*S. amplexicaulis* (b)	1.63 ± 0.00 ^c^ (6.58)	4.60 ± 0.01 ^c^ (18.58)	0.50 ± 0.00 ^cg^ (2.02)	0.42 ± 0.01 ^c^ (1.70)
*S. amplexicaulis* (l)	1.14 ± 0.01 ^d^ (3.43)	10.60 ± 0.01 ^d^ (31.88)	1.37 ± 0.01 ^d^ (4.12)	0.63 ± 0.00 ^d^ (1.89)
*S. babylonica* (b)	1.95 ± 0.02 ^e^ (10.34)	1.40 ± 0.01 ^e^ (7.42)	0.38 ± 0.00 ^abcg^ (2.01)	0.36 ± 0.01 ^e^ (1.91)
*S. babylonica* (l)	0.93 ± 0.00 ^f^ (3.11)	1.02 ± 0.04 ^f^ (3.41)	0.27 ± 0.00 ^ab^ (0.90)	0.36 ± 0.00 ^e^ (1.21)
*S. fragilis* (b)	1.22 ± 0.00 ^g^ (6.67)	1.01 ± 0.02 ^f^ (5.52)	0.32 ± 0.01 ^abc^ (1.75)	0.37 ± 0.00 ^f^ (2.02)
*S. fragilis* (l)	2.23 ± 0.01 ^h^ (8.00)	2.10 ± 0.05 ^g^ (7.53)	0.81 ± 0.00 ^e^ (2.91)	0.46 ± 0.01 ^g^ (1.65)
*S. purpurea* (b)	1.88 ± 0.02 ^i^ (9.84)	2.50 ± 0.02 ^h^ (13.08)	0.48 ± 0.36 ^bcg^ (2.51)	0.56 ± 0.00 ^h^ (2.93)
*S. purpurea* (l)	1.80 ± 0.01 ^j^ (5.52)	6.80 ± 0.02 ^i^ (20.85)	0.72 ± 0.01 ^ef^ (2.21)	0.98 ± 0.01 ^i^ (3.00)
*S. triandra* (b)	1.29 ± 0.01 ^k^ (8.00)	1.02 ± 0.04 ^f^ (6.33)	0.72 ± 0.01 ^ef^ (4.47)	0.45 ± 0.00 ^g^ (2.79)
*S. triandra* (l)	1.73 ± 0.01 ^l^ (6.36)	2.02 ± 0.05 ^j^ (7.43)	0.59 ± 0.01 ^cefg^ (2.17)	0.37 ± 0.00 ^f^ (1.36)

Data are presented as means of triplicate measurements ± SD. Different superscript letters within the same column indicate significant differences between means at the 0.05 level. Data in parentheses are presented as means of triplicate measurements.

**Table 4:** Antioxidant activity of ultrasonic extracts of *Salix* bark (b) and leaves (l) measured with DPPH^•^ and ^•^OH scavenging assays.

Species	DPPH^•^	^•^OH
	IC_50_ (µg/mL) ^*^	
*S. alba* (b)	3.32 ± 0.08 ^a^	17.08 ± 0.26
*S. alba* (l)	12.25 ± 0.25 ^b^	15.35 ± 0.15
*S. amplexicaulis* (b)	8.50 ± 0.11 ^c^	33.24 ± 0.20
*S. amplexicaulis* (l)	11.49 ± 0.12 ^d^	20.74 ± 0.16
*S. babylonica* (b)	4.29 ± 0.07 ^e^	36.28 ± 0.23
*S. babylonica* (l)	44.31 ± 0.33 ^f^	28.49 ± 0.11
*S. fragilis* (b)	18.44 ± 0.14 ^g^	18.44 ± 0.15
*S. fragilis* (l)	11.91 ± 0.09 ^h^	23.79 ± 0.11
*S. purpurea * (b)	5.68 ± 0.08 ^i^	24.26 ± 0.14
*S. purpurea* (l)	13.19 ± 0.10 ^j^	27.22 ± 0.14
*S. triandra* (b)	4.32 ± 0.08 ^e^	26.77 ± 0.15
*S. triandra* (l)	4.36 ± 0.14 ^e^	32.64 ± 0.15

* Data are means of triplicate measurements ± SD. Different superscript letters within the same column (DPPH^•^) or their absence (^•^OH) indicate significant differences between means at the 0.05 level.

**Table 5:** Results of molecular docking analysis of active compounds against TNF-α.

Compound	Binding Energy (kcal/mol)	Interaction Site	
		Hydrogen Bond	Pi–Pi /Pi–Alkyl /Pi–Sigma
Chlorogenic acid	−7.55	GlyA 121; SerB 60; LysB 98; ProB 117; TyrB 119; LeuB 120; TyrB 151	^b^ TyrB 119
Naringenin	−6.93	SerA 60; GlnA 61; TyrA 151; SerB 60; LeuB 120	^a^ TyrA 119; ^a^ Tyr B 119
Epicatechin	−6.21	GlnA 61; GlyA 121; SerB 60; LeuB 120; TyrB 151	^a^ TyrA 119; ^ab^ TyrB 119
Quercetin	−6.14	GlyA 121; GlyB 121	^a^ TyrA 119
Rutin	−5.71	GlyA 121; SerB 60; TyrB 119; LeuB 120; TyrB 151; GlnD 125	^b^ LeuA 57; ^b^ TyrA 59; ^b^ LeuD 55
*p*-coumaric acid	−5.29	TyrB 119; GlyB 121	^b^ AlaB 96; ^c^ LeuB 120
*p*-hydoxybenzoic acid	−5.22	LysA 98; TyrA 119; TyrB 119	-
Syringic acid	−5.18	LysA 98; ProB 117; TyrB 119	^b^ AlaB 96; ^b^ LysB 98; ^b^ IleB 118; ^b^ TyrB 119
Gallic acid	−5.00	LysA 98; ProB 117; TyrB 119	^a^ TyrB 119
Salicin	−4.94	SerA 60; GlnA 61; LeuA 120; GlyA 121; TyrA 151	^a^ TyrB 59
*trans*-cinnamic acid	−4.86	TyrB 119	^b^ LeuB 94; ^b^ LeuD 55; ^c^ LeuB 120
Acetylsalicylic acid	−5.83		

^a^ Pi–Pi, ^b^ Pi–Alkyl, ^c^ Pi–Sigma

**Table 6:** Results of molecular docking analysis of active compounds against interleukin 6.

Compound	Binding Energy (kcal/mol)	Interaction Site	
		Hydrogen Bond	Pi–Alkyl /Alkyl /Pi–Sigma /Pi–Lone Pair /Pi–Cation
Chlorogenic acid	−6.51	Asp 34; Arg 182	^a^ Leu 33; ^b^ Leu 178; ^b^ Arg 179
*p*-coumaric acid	−6.45	Asp 26; Arg 30; Arg 179; Arg 182	^a^ Leu 178
Quercetin	−6.01	Lys 66; Met 67; Cys 73; Gln 183	^d^ Ser 176; ^e^ Arg 179
*p*-hydoxybenzoic acid	−5.87	Asp 26; Arg 30; Arg 179; Arg 182	^a^ Leu 178
Gallic acid	−5.81	Asp 26; Arg 30; Arg 179; Gln 175; Arg 182	^c^ Leu 178; ^a^ Arg 182
Naringenin	−5.65	Asp 34; Gln 175; Arg 182	^a^ Arg 30; ^a^ Leu 33; ^c^ Leu 178
Epicatechin	−5.56	Asp 26; Arg 30; Asp 34; Gln 175	^c^ Leu 33; ^a^ Lys 171; ^c^ Leu 178
*trans*-cinnamic acid	−5.40	Arg 179; Arg 182	^c^ Leu 178
Syringic acid	−4.18	Asp 26; Arg 182	^c^ Leu 178
Salicin	−3.96	Asp 34; Gln 175	^a^ Lys 171
Rutin	−3.57	Arg 30; Gln 175; Arg 179; Arg 182	^a^ Arg 30; ^c^ Leu 33; ^ac^ Leu 178; ^ab^ Arg 179
Acetylsalicylic acid	−4.66		

^a^ Pi–Alkyl, ^b^ Alkyl, ^c^ Pi–Sigma, ^d^ Pi–Lone Pair, ^e^ Pi–Cation.

## Data Availability

Data will be made available upon request.

## References

[1] Siebert S., Tsoukas A., Robertson J., McInnes I. (2015). Cytokines as Therapeutic Targets in Rheumatoid Arthritis and Other Inflammatory Diseases. Pharmacol. Rev..

[2] Salman H.A., Yaakop A.S., Aladaileh S., Mustafa M., Gharaibeh M., Kahar U.M. (2023). Inhibitory effects of Ephedra alte on IL-6, hybrid TLR4, TNF-α, IL-1β, and extracted TLR4 receptors: In silico molecular docking. Heliyon.

[3] Khayyal M.T., El-Ghazaly M.A., Abdallah D.M., Okpanyi S.N., Kelber O., Weiser D. (2005). Mechanisms Involved in the Anti-inflammatory Effect of a Standardized Willow Bark Extract. Arzneimittelforschung.

[4] Lobo V., Patil A., Phatak A., Chandra N. (2010). Free radicals, antioxidants and functional foods: Impact on human health. Pharmacogn. Rev..

[5] Zamudio-Cuevas Y., Martínez-Flores K., Martínez-Nava G.A., Clavijo-Cornejo D., Fernández-Torres J., Sánchez-Sánchez R. (2022). Rheumatoid arthritis and oxidative stress, a review of a decade. Cell. Mol. Biol..

[6] Wang X., Fan D., Cao X., Ye Q., Wang Q., Zhang M., Xiao C. (2022). The Role of Reactive Oxygen Species in the Rheumatoid Arthritis-Associated Synovial Microenvironment. Antioxidants.

[7] López-Armada M.J., Fernández-Rodríguez J.A., Blanco F.J. (2022). Mitochondrial Dysfunction and Oxidative Stress in Rheumatoid Arthritis. Antioxidants.

[8] Gligorić E., Igić R., Čonić B.S., Kladar N., Teofilović B., Grujić N. (2023). Chemical profiling and biological activities of “green” extracts of willow species (*Salix* L., Salicaceae): Experimental and chemometric approaches. Sustain. Chem. Pharm..

[9] Schmid B., Kötter I., Heide L. (2001). Pharmacokinetics of salicin after oral administration of a standardised willow bark extract. Eur. J. Clin. Pharmacol..

[10] Bonaterra G., Heinrich E., Kelber O., Weiser D., Metz J., Kinscherf R. (2010). Anti-inflammatory effects of the willow bark extract STW 33-I (Proaktiv®) in LPS-activated human monocytes and differentiated macrophages. Phytomedicine.

[11] Bonaterra G.A., Kelber O., Weiser D., Metz J., Kinscherf R. (2010). In vitro anti-proliferative effects of the willow bark extract STW 33-I. Arzneimittelforschung.

[12] Nahrstedt A., Schmidt M., Jäggi R., Metz J., Khayyal M.T. (2007). Willow bark extract: The contribution of polyphenols to the overall effect. Wien. Med. Wochenschr..

[13] Gligorić E., Igić R., Suvajdžić L., Grujić-Letić N. (2019). Species of the Genus *Salix* L.: Biochemical Screening and Molecular Docking Approach to Potential Acetylcholinesterase Inhibitors. Appl. Sci..

[14] Tawfeek N., Mahmoud M.F., Hamdan D.I., Sobeh M., Farrag N., Wink M., El-Shazly A.M. (2021). Phytochemistry, Pharmacology and Medicinal Uses of Plants of the Genus Salix: An Updated Review. Front. Pharmacol..

[15] Vera J., Herrera W., Hermosilla E., Díaz M., Parada J., Seabra A.B., Tortella G., Pesenti H., Ciudad G., Rubilar O. (2023). Antioxidant Activity as an Indicator of the Efficiency of Plant Extract-Mediated Synthesis of Zinc Oxide Nanoparticles. Antioxidants.

[16] Stefanucci A., Scioli G., Marinaccio L., Zengin G., Locatelli M., Tartaglia A., Della Valle A., Cichelli A., Novellino E., Pieretti S. (2022). A Comparative Study on Phytochemical Fingerprint of Two Diverse *Phaseolus vulgaris* var. Tondino del Tavo and Cannellino Bio Extracts. Antioxidants.

[17] Pobłocka-Olech L., Krauze-Baranowska M., Głód D., Kawiak A., Łojkowska E. (2010). Chromatographic analysis of simple phenols in some species from the genus *Salix*. Phytochem. Anal..

[18] Enayat S., Banerjee S. (2009). Comparative antioxidant activity of extracts from leaves, bark and catkins of *Salix aegyptiaca* sp. Food Chem..

[19] Mollica A., Zengin G., Sinan K.I., Marletta M., Pieretti S., Stefanucci A., Etienne O.K., Jekő J., Cziáky Z., Bahadori M.B. (2022). A Study on Chemical Characterization and Biological Abilities of *Alstonia boonei* Extracts Obtained by Different Techniques. Antioxidants.

[20] Lee J.E., Noh S.-K., Kim M.J. (2022). Effects of Enzymatic- and Ultrasound-Assisted Extraction on Physicochemical and Antioxidant Properties of Collagen Hydrolysate Fractions from Alaska Pollack (*Theragra chalcogramma*) Skin. Antioxidants.

[21] Gligorić E.I., Igić R., Suvajdžić L.Đ., Teofilović B.D., Grujić-Letić N.N. (2020). *Salix eleagnos* Scop.—A novel source of antioxidant and anti-inflammatory compounds: Biochemical screening and in silico approaches. S. Afr. J. Bot..

[22] Zia K., Ashraf S., Jabeen A., Saeed M., Nur-E-Alam M., Ahmed S., Al-Rehaily A.J., Ul-Haq Z. (2020). Identification of potential TNF-α inhibitors: From in silico to in vitro studies. Sci. Rep..

[23] Tran Q.-H., Nguyen Q.-T., Vo N.-Q., Mai T.T., Tran T.-T., Tran T.-D., Le M.-T., Trinh D.-T.T., Thai K.-M. (2022). Structure-based 3D-Pharmacophore modeling to discover novel interleukin 6 inhibitors: An in silico screening, molecular dynamics simulations and binding free energy calculations. PLoS ONE.

[24] Shirsath S.R., Sonawane S.H., Gogate P.R. (2012). Intensification of extraction of natural products using ultrasonic irradiations—A review of current status. Chem. Eng. Process..

[25] Aliaño-González M.J., Barea-Sepúlveda M., Espada-Bellido E., Ferreiro-González M., López-Castillo J.G., Palma M., Barbero G.F., Carrera C. (2022). Ultrasound-Assisted Extraction of Total Phenolic Compounds and Antioxidant Activity in Mushrooms. Agronomy.

[26] Ostolski M., Adamczak M., Brzozowski B., Wiczkowski W. (2021). Antioxidant Activity and Chemical Characteristics of Supercritical CO_2_ and Water Extracts from Willow and Poplar. Molecules.

[27] Aware C.B., Patil R.R., Vyavahare G.D., Gurme S.T., Jadhav J.P. (2019). Ultrasound-Assisted Aqueous Extraction of Phenolic, Flavonoid Compounds and Antioxidant Activity of Mucuna macrocarpa Beans: Response Surface Methodology Optimization. J. Am. Coll. Nutr..

[28] Nyman T., Julkunen-Tiitto R. (2005). Chemical variation within and among six northern willow species. Phytochemistry.

[29] Jürgenliemk G., Petereit F., Nahrstedt A. (2007). Flavan-3-ols and procyanidins from the bark of *Salix purpurea* L. Pharmazie.

[30] European Medicines Agency (2017). Assessment Report on Salix [Various Species Including S. purpurea L., S. daphnoides Vill., S. fragilis L.], Cortex.

[31] Dickmann D.I., Kuzovkina J., Isebrands J.G., Richardson J. (2014). Poplars and Willows of the World, with Emphasis on Silviculturally Important Species. Poplars and Willows: Trees for Society and the Environment.

[32] Tsiaka T., Kritsi E., Bratakos S.M., Sotiroudis G., Petridi P., Savva I., Christodoulou P., Strati I.F., Zoumpoulakis P., Cavouras D. (2023). Quality Assessment of Ground Coffee Samples from Greek Market Using Various Instrumental Analytical Methods, In Silico Studies and Chemometrics. Antioxidants.

[33] Lu H., Tian Z., Cui Y., Liu Z., Ma X. (2020). Chlorogenic acid: A comprehensive review of the dietary sources, processing effects, bioavailability, beneficial properties, mechanisms of action, and future directions. Compr. Rev. Food Sci. Food Saf..

[34] Odunola O., Olugbami J.O., Gbadegesin M.A. (2015). In vitro free radical scavenging and antioxidant properties of ethanol extract of Terminalia glaucescens. Pharmacogn. Res..

[35] Treml J., Šmejkal K. (2016). Flavonoids as Potent Scavengers of Hydroxyl Radicals. Compr. Rev. Food Sci. Food Saf..

[36] Zhao H., Wu L., Yan G., Chen Y., Zhou M., Wu Y., Li Y. (2021). Inflammation and tumor progression: Signaling pathways and targeted intervention. Signal Transduct. Target. Ther..

[37] Ivanova L., Karelson M. (2022). The Impact of Software Used and the Type of Target Protein on Molecular Docking Accuracy. Molecules.

[38] Nayak B.N., Buttar H.S. (2016). Evaluation of the antioxidant properties of tryptophan and its metabolites in in vitro assay. J. Complement. Integr. Med..

[39] Guvenc A., Arihan O., Altun M., Dinc E., Baleanu D. (2007). Determination of salicin content of some *Salix* L. species by HPLC method. Rev. Chim..

[40] Hanwell M.D., Curtis D.E., Lonie D.C., Vandermeersch T., Zurek E., Hutchison G.R. (2012). Avogadro: An advanced semantic chemical editor, visualization, and analysis platform. J. Cheminform..

[41] He M.M., Smith A.S., Oslob J.D., Flanagan W.M., Braisted A.C., Whitty A., Cunningham B.C. (2005). Small-molecule inhibition of TNF-α. Science.

[42] Somers W., Stahl M., Seehra J.S. (1997). 1.9 Å crystal structure of interleukin 6: Implications for a novel mode of receptor dimerization and signaling. EMBO J..

[43] Morris G.M., Huey R., Lindstrom W., Sanner M.F., Belew R.K., Goodsell D.S., Olson A.J. (2009). AutoDock4 and AutoDockTools4: Automated docking with selective receptor flexibility. J. Comput. Chem..

